# Gelatin–Chitosan Hydrogel Biological, Antimicrobial and Mechanical Properties for Dental Applications

**DOI:** 10.3390/biomimetics8080575

**Published:** 2023-12-01

**Authors:** Andrea Itzamantul Flores-Espinoza, Rene Garcia-Contreras, Dulce Araceli Guzman-Rocha, Benjamin Aranda-Herrera, Patricia Alejandra Chavez-Granados, Carlos A. Jurado, Yasser F. Alfawaz, Abdulrahman Alshabib

**Affiliations:** 1 Interdisciplinary Research Laboratory (LII), Nanostructures and Biomaterials Area, National School of Higher Studies (ENES), Leon Unit, National Autonomous University of Mexico (UNAM), Leon 37689, Mexico; andflor320@gmail.com (A.I.F.-E.); rgarciac@enes.unam.mx (R.G.-C.); dulguzman30@gmail.com (D.A.G.-R.); benja.aherrera@gmail.com (B.A.-H.); chgranadosalejandra@gmail.com (P.A.C.-G.); 2 Department of Prosthodontics, The University of Iowa College of Dentistry and Dental Clinics, Iowa City, IA 52242, USA; carlos-jurado@uiowa.edu; 3 Department of Restorative Dentistry, King Saud University College of Dentistry, Riyadh 11545, Saudi Arabia; yalfawaz@ksu.edu.sa

**Keywords:** gelatin–chitosan hydrogel, 3D cell culture, antimicrobial, FTIR, mechanical properties

## Abstract

Chitosan, a natural polysaccharide sourced from crustaceans and insects, is often used with hydrogels in wound care. Evaluating its cytotoxicity and antimicrobial properties is crucial for its potential use in dentistry. Objective: To investigate the mechanical properties of gelatin hydrogels based on decaethylated chitosan and antimicrobial activity against *Streptococcus mutans* and their biological effects with stem cells from apical papilla (SCAPs). Material and methods: Gelatin–chitosan hydrogels were synthesized at concentrations of 0%, 0.2% and 0.5%. Enzymatic and hydrolytic degradation, along with swelling capacity, was assessed. Fourier transform infrared spectroscopy (FTIR) analysis was employed to characterize the hydrogels. The interaction between hydrogels and SCAPs was examined through initial adhesion and cell proliferation at 24 and 48 h, using the Thiazolyl Blue Tetrazolium Bromide (MTT assay). The antimicrobial effect was evaluated using agar diffusion and a microdilution test against *S. mutans*. Uniaxial tensile strength (UTS) was also measured to assess the mechanical properties of the hydrogels. Results: The hydrogels underwent hydrolytic and enzymatic degradation at 30, 220, 300 min and 15, 25, 30 min, respectively. Significantly, (*p* < 0.01) swelling capacity occurred at 20, 40, 30 min, respectively. Gelatin–chitosan hydrogels’ functional groups were confirmed using vibrational pattern analysis. SCAPs proliferation corresponded to 24 h = 73 ± 2%, 82 ± 2%, 61 ± 6% and 48 h = 83 ± 11%, 86 ± 2%, 44 ± 2%, respectively. The bacterial survival of hydrogel interaction was found to be 96 ± 1%, 17 ± 1.5% (*p* < 0.01) and 1 ± 0.5% (*p* < 0.01), respectively. UTS showed enhanced (*p* < 0.05) mechanical properties with chitosan presence. Conclusion: Gelatin–chitosan hydrogels displayed favorable degradation, swelling capacity, mild dose-dependent cytotoxicity, significant proliferation with stem cells from apical papilla (SCAPs), substantial antimicrobial effects against *S. mutans* and enhanced mechanical properties. These findings highlight their potential applications as postoperative care dressings.

## 1. Introduction

Cell culture has been referred to as the in vitro maintenance and cultivation of cells, tissues, or organs obtained from animals or plants, and this process entails extracting cells from their natural tissues and introducing them into an artificial environment conducive to controlled growth, replication, and metabolic sustenance [1]. Various categories of cell cultures are currently available and each of them serve distinct purposes. In contemporary times, cell cultures play a pivotal role in assessing the effectiveness and cytotoxicity of medical substances, facilitating protein expression and investigating the dynamics between pathogens and hosts.

Cell culture techniques have been performed for several years, with traditional two-dimensional (2D) culturing aiming to replicate the intricate dynamics of the human body [2,3]. Moreover, the introduction of the transwell culture system, a layered 2D approach, sought to emulate in vivo conditions through co-culturing. However, this method may display some limitations in sustaining cellular functionality over extended durations [4,5], and nowadays 3D cell culture development has emerged as a solution to enhance culture efficiency and cellular activities. Biomaterials come in diverse forms, including hydrogels, solid scaffolds, decellularized native tissue, and surfaces with ultra-low attachment [6,7]. The novel workflows with 3D culture methodologies have made remarkable strides, giving rise to a plethora of applications and advancements.

Chitosan, which has been shown to have bioadhesive properties, biocompatibility, and antibacterial activity, has also been considered an option to act with an oral environment [8,9,10]. Chitosan has demonstrated no antibacterial activity against both gram-negative and gram-positive bacteria. Gram-negative bacteria have been used in antibacterial studies including *Escherichia coli* (*E. coli*), *Porphyromonas gingivalis* (*P. gingivalis*), *Klebsiella pneumoniae*, *Pseudomona aeruginosa*, *Salmonella,* and *Serratia marcescensy*. Gram-positive bacteria including *Streptococcus mutans* (*S. mutans*), *Staphylococcus aureus* (*S. aureus*), and *Bacillus* are the most commonly used [11,12]. It has been proven that chitosan has biodegradable and non-toxic properties, and it helps in adhering to mammalian cells. Furthermore, studies have reported that chitosan also helps in the bone formation and it consequently increases the formation of osteoblasts [13]. In addition to all the above-mentioned characteristics, chitosan can be combined with other compounds to produce various products such as hydrogels, resins, sponges, pastes, membranes, and fibers [14].

Understanding hydrogel structures and their smart gelation process is crucial for tailoring hydrogels with specific characteristics [15,16]. Chitosan is a well-known key component in hydrogels, due to its biocompatibility, biodegradability, and minimal toxicity, making it an ideal choice for producing innovative biomaterials. Furthermore, its biocompatibility has been extensively researched and proven to be successful in animal studies [17].

Chitosan-based hydrogels are generally reversible at a certain temperature and sensitive to pH as they swell in acidic pH and contract in basic media. Due to its exceptional properties, this biopolymer is a superb option for various applications, including drug administration, dynamic phototherapy, and blood anticoagulation [18,19].

The study proposed that gelatin–chitosan hydrogels could potentially be used in various dental applications such as offering enhanced interaction, cell proliferation, antibacterial properties, and appropriate degradation. This study aimed to evaluate the applicability of gelatin–chitosan hydrogels in enhancing the interaction and proliferation of stem cells from the apical papilla (SCAPs), its antibacterial efficacy against *Streptococcus mutans*, composition, hydrolytic and enzymatic degradation, swelling characteristics, and ultimately its uniaxial tensile strength (UTS) properties.

## 2. Materials and Methods

### 2.1. Materials and Equipment

Acetic acid 99% (Sigma-Aldrich, Saint Louis, MO, USA), agitation incubator (VORTEMP 1550 LABNET, Edison, NJ, USA), autoclave (Tuttnauer, Hauppage, New York, NY, USA), analytical balance (Denver instrument, Arvada, CO, USA), deacetylated chitosan (448869-250G; low molecular weight, deacetylation degree ≥75%, Sigma-Aldrich, Saint Louis, MO, USA), deionized water (Karal, Leon, Gto, Mexico), densitometer (Grant-bio, Grant Instruments, Cambridge, UK), distilled water (Karal, Leon, Gto, Mexico), gelatin from porcine skin gel 300, type A. (G2500-500G; Sigma-Aldrich, Saint Louis, MO, USA), minimum essential medium eagle medium (MEM, Sigma-Aldrich, Saint Louis, MO, USA), fetal bovine serum (FBS, Gibco, USA), 1% glutamine (Gibco, Carlsbad, CA, USA), and 1% antibiotic (PenStrep, Sigma-Aldrich, Saint Louis, MO, USA), Thiazolyl Blue Tetrazolium Bromide (MTT method, Sigma-Aldrich, Saint Louis, MO, USA), 10 cm and 24-well Petri culture plates (Corning, Tewksbury, MA, USA), VORTEMP 1550 centrifuge (LABNET, Edison, NJ, USA), bacteria incubator (Incucell, Planegg, Germany), Coomassie blue (BIO-RAD, Hercules, CA, USA), flow hood for bacteria (Thermo Scientific, Grand Island, NY, USA), horizontal flow hood (Biobase, Wolfenbüttel, Germany).

### 2.2. Gelatin–Chitosan Hydrogel Preparation

The method for the preparation of the gelatin–chitosan hydrogel was carried out using the physical method of crosslinking. Given the ionic nature of these polymers, there is an electrostatic interaction between chitosan amino groups and the gelatin carboxyl group. Initially, a 1% chitosan solution was prepared by adding 9.9 mL of distilled water to a beaker. Subsequently, 100 μL of 1% acetic acid and 0.1 g of deacetylated chitosan sourced from shrimp shells were introduced. Slow and continuous stirring facilitated the complete dissolution of chitosan powder over a 24 h period, resulting in increased viscosity and a noticeable color change. The hydrogel preparation consisted of two phases. In the first phase, a 1% chitosan solution (*w*/*v*) and a 15% porcine skin gelatin solution (*w*/*v*) were meticulously prepared at 80 °C. This gelation process required thorough mixing to ensure homogeneity while maintaining a temperature range of 60–80 °C. In the subsequent phase, these solutions were combined and poured into a Petri dish, serving as a mold for the hydrogel. Three hydrogel variations were synthesized: one without chitosan and two others with chitosan at concentrations of 0.2% and 0.5%. The necessary chitosan quantity was added after complete gelatin dissolution to reach the intended concentration. After achieving complete dissolution of all hydrogel components, the mixture underwent autoclave sterilization before being poured into 24-well culture dishes, which were prepared for the subsequent cell inoculation. Samples were then extracted from the molded hydrogel using a 5 × 2 mm punch (Integra Miltex, Princeton, NJ, USA).

### 2.3. Hydrolytic and Enzymatic Degradation

Samples were stored in Petri dishes, corresponding to different concentrations (0%, 0.2%, and 0.5% of chitosan). They were weighed using an analytical balance before undergoing the degradation process. The initial weight was recorded and compared with the weight at intervals of 20 min (for hydrolytic degradation) and every 5 min (for enzymatic degradation) until complete degradation was achieved. Subsequently, samples from each group were placed in 1.5 mL Eppendorf tubes (Sigma-Aldrich, Saint Louis, MO, USA) together with a solution of PBS or 0.5% trypsin (Sigma-Aldrich, Saint Louis, MO, USA) for hydrolytic and enzymatic degradation, respectively. These samples in the solution-containing tubes were agitated within an incubator at 180 rpm and a temperature of 37 °C. Agitation was paused for the specified intervals. During these pauses, the hydrogels were extracted, weighed, and their weights were recorded. To validate these results, an analysis was conducted by measuring the absorbance of the solutions using a UV-VIS spectrophotometer (Multiskan go, Thermo-Scientific, Vantaa, Finland) at 390 nm. Samples of the PBS and trypsin solution, into which the various hydrogel groups were immersed, were transferred using a serological pipette to a 96-well plate with 50 µL of the solutions per well.

### 2.4. Gelatin–Chitosan Hydrogel Characterization Using FTIR

The hydrogels samples (0%, 0.2%, and 0.5% of chitosan) of 5 × 2 mm were analyzed using Fourier transform infrared spectroscopy (FTIR, PerkinElmer, McPherson St. Markham, ON, Canada) using an accessory of Attenuated Total Reflection (ATR) for the interaction between infrared radiation and the sample to be analyzed. The samples were freeze-dried to remove excess water for further analysis. The FTIR spectra were measured in transmittance units spanning the range of 4000–400 cm^−1^.

### 2.5. Gelatin–Chitosan Hydrogel Swelling

The hydrogel samples of 5 × 2 mm were tested for swelling capacity, which was carried out through initial weighing using an analytical balance. Following that, swelling procedures were initiated by placing individual samples in 1.5 mL Eppendorf tubes, each filled with 1 mL of distilled water at a temperature of 37 °C. The incubation was interrupted every 10 min for the measurement of the weight samples. This process was repeated until the hydrogel reached its maximum water absorption form 0–60 min. The experiments were conducted in triplicate with *n* = 9. The swelling values obtained were fitted to a Voigt mathematical model.

### 2.6. SCAPs Cell Culture and Characterization

The process of isolating, cultivating, and characterizing of SCAPs underwent a comprehensive review and gained approval from the bioethics committee at the ENES Leon Unit, UNAM, authorized under code CE_16 004_SN. The study involved the utilization of partially erupted third molars with incomplete apical formation, earmarked for odontectomy procedures within the ENES Leon Unit clinics. These molars were obtained from patients aged 16, and thorough assessment ensured the absence of both pulpal and periapical pathology. Under the controlled conditions of a horizontal laminar flow hood, the meticulous extraction of apical papilla tissue was conducted. After the tissue extraction, explants measuring 1 × 1 mm were meticulously prepared. These explants were subsequently placed in a Petri dish which had a diameter of 10 cm, fully immersed within a culture medium consisting of minimum essential medium eagle medium (MEM) enriched with 20% fetal bovine serum (FBS), 1% glutamine, and 1% antibiotic. The cultures were then upheld at a temperature of 37 °C, with a CO_2_ concentration of 5% and humidity maintained at 95%, for an interval extending to 21 days.

The SCAPs displayed a fibroblastoid morphology, adhered to the plate, and reached a confluence of 90%. Subsequent subcultures were cultivated using MEM medium supplemented with 10% FBS, glutamine, and antibiotics. The cellular characterization process was conducted after five cell divisions [equivalent to 5 population doubling levels (PDL)]. This characterization was further supported by immunocytochemistry utilizing antibodies against vimentin and CD 56 (Sigma-Aldrich, Saint Louis, MO, USA) and observed using optical microscopy (Leica, Wetzlar, Germany) at 20× and 40× magnification.

### 2.7. Gelatin–Chitosan Hydrogel SCAPs Interaction and Proliferation

To assess this interaction, a cell subculture was performed using the drop method. Hydrogel discs were meticulously placed on a microscope slide, and SCAPs were cultured on their surface at a concentration of 1 × 10^6^ cells/mL (30 µL). Subsequently, the cultures were incubated at 37 °C with a 5% CO_2_ concentration and 95% humidity. This incubation lasted for one hour to facilitate cell interaction, or alternatively, for 24 and 48 h to observe cell proliferation.

For the cell interaction analysis, the hydrogel discs were positioned on a microscope slide and firmly fixed using a 4% formaldehyde solution mixed in a 1:1 ratio with PBS. This fixing solution was allowed to interact for 15 min. Subsequently, the formaldehyde solution was removed, and the samples were subjected to dehydration using a series of ethanol gradients (25%, 75%, and 100%), with each step lasting 5 min. Following dehydration, the samples were stained with a Coomassie blue solution for 15 min and washed two times with PBS. Finally, the samples were covered with an additional microscope slide, enabling the upper surface of the hydrogels to be observed using optical microscopy (Leica, Wetzlar, Germany) at 40× magnification.

To evaluate cell proliferation, cell viability was assessed using the MTT method [7]. Briefly, cells were cultivated for a duration of 7 h with a concentration of 0.2 mg/mL (Thiazolyl Blue Tetrazolium Bromide, MTT assay) in fresh MEM supplemented with 10% FBS. The formazan compound generated during the incubation was dissolved by adding 0.1 mL of dimethyl sulfoxide (DMSO, Karal, Leon, Guanajuato, Mexico). The absorbance of the solution obtained was assessed at a wavelength of 570 nm employing a microplate spectrophotometer reader. The assessment of cytotoxicity adhered to the guidelines outlined in ISO 10993-5:2009, tests for the in vitro cytotoxicity of medical devices.

### 2.8. Antimicrobial Activity of Gelatin–Chitosan Hydrogels

For the Kirby–Bauer agar diffusion and broth microdilution assays, *Streptococcus mutans* (ATCC 36668) was selected as the experimental bacteria. The bacteria were inoculated onto Mueller–Hinton agar plates (BD Bioxon, Mexico City, Mexico) and incubated at 37 °C for 24 h. In brief, six uniform colonies were transferred into 15 mL of the Mueller–Hinton broth, followed by an incubation period of 24 h at 37 °C. The bacterial culture was standardized to a concentration of 0.5 on the McFarland scale, corresponding to approximately 1 × 10^8^ CFU/mL. Subsequently, 30 µL of this standardized culture was utilized to create a working solution through a final dilution of 1:1000. For the agar diffusion test, agar was punctured with a cylindrical blade (5 × 2 mm), and gelatin–chitosan hydrogels (0%, 0.2%, 0.5%) were introduced into the cavities. These plates were then incubated at 37 °C for 24 h to assess inhibition zones measured in millimeters. Regarding the broth microdilution assay, the gelatin–chitosan hydrogel was placed at the bottom of 96-well plates prior to bacterial inoculation, then the plates were incubated for 24 h. The number of viable bacteria was evaluated employing 0.2 mg/mL of MTT. Following this, the blend was incubated in the absence of light for 4 h at room temperature. Afterward, the microplate was examined using a microplate spectrophotometer reader at a wavelength of 595 nm. To ensure internal validity, a sterile saline solution served as the negative control, while 0.12% chlorhexidine (Dentscare Ltda, FGM, Joinville, Santa Catarina, Brazil) acted as the positive control. Data analysis adhered to the guidelines outlined by the Clinical and Laboratory Standards Institute (CLSI).

### 2.9. Gelatin–Chitosan Hydrogel Uniaxial Tensile Strength (UTS)

The hydrogels were evaluated using a uniaxial tensile test (UTS). The hydrogels were evaluated following the ASTM D 882-02 standard for assessing the tensile properties. The testing was carried out using a universal testing machine (Kejian Instrument Co., Ltd., Dongguan, Guangdong, China), with the initial scaffold area measuring 20 × 10 × 5 mm positioned between clamps. A crosshead speed of 1 mm/min was utilized until failure occurred. Following this, the tensile strength was calculated by dividing the force applied to the sample (measured in Newtons) by the sample’s cross-sectional area (in mm) and expressed in megapascals (MPa). The experiment involved a sample size of *n* = 6 per group.

### 2.10. Statistical Analysis

The experiments were performed in triplicate across three independent trials to ensure reproducibility (*n* = 9). The resulting data were presented in terms of the mean, standard deviation, and percentages. For statistical analysis, the normality of the data was evaluated through the Shapiro–Wilk normality test, followed by Tukey’s post hoc analysis using ANOVA. The significance was set at a threshold of *p* < 0.05, and a 95% confidence interval was established.

## 3. Results

### 3.1. Degradation of the Gelatin–Chitosan Hydrogels

**Figure 1:**
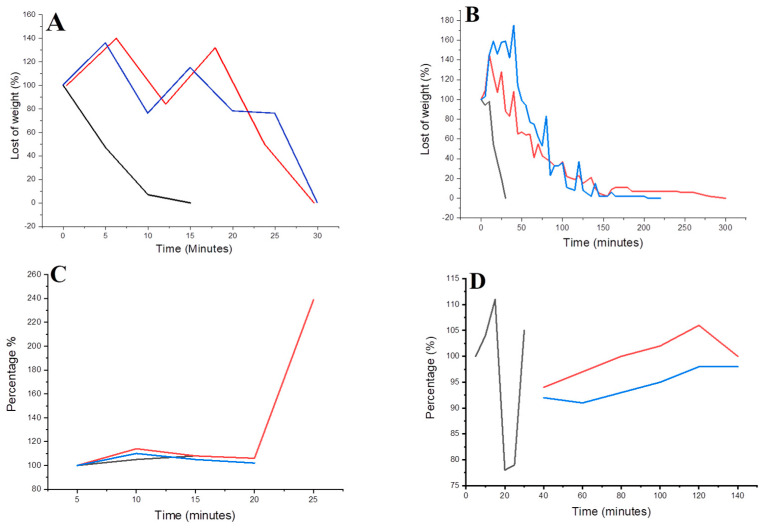
Enzymatic and hydrolytic degradation of the gelatin–chitosan hydrogels. Gelatin–chitosan hydrogels (5 × 2 mm) were weighed, placed in Eppendorf tubes, and incubated with PBS and trypsin at 37 °C, 180 rpm in an agitated incubator. The hydrogels were weighed (**A**,**B**), and their absorbance was measured at 390 nm using a UV-VIS spectrophotometer (**C**,**D**). [(**A**,**C**) enzymatic degradation, (**B**,**D**) hydrolytic degradation]. Data represent mean values. The black line corresponds to the control group (gelatin hydrogel), whereas the blue and red lines depict the experimental group with gelatin–chitosan hydrogel at 0.2% and 0.5%, respectively.

Degradation tests showed that the (i) 0%, (ii) 0.2% and (iii) 0.5% gelatin–chitosan hydrogels degrade completely within (i) 15 and 30 min, (ii) 25 min and 220 min, (iii) 30 min and 350 min for enzymatic and hydrolytic degradation, respectively. The degradation process was validated using exponential absorbance data, wherein an increase in absorbance aligned with the weight loss, signifying a direct correlation between increased medium degradation and absorbance growth. Figure 1 shows the results of enzymatic and hydrolytic degradation.

### 3.2. Gelatin–Chitosan Hydrogel Characterization Using FTIR

**Figure 2:**
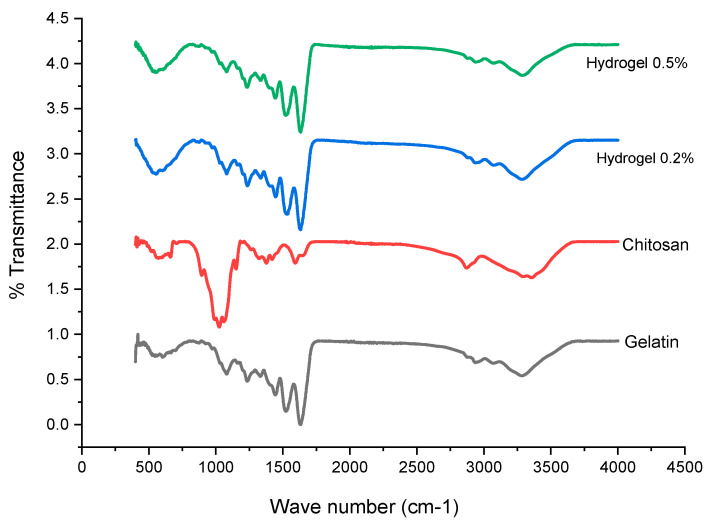
Gelatin–chitosan hydrogels characterization using Fourier transform infrared spectroscopy (FTIR). Hydrogels (5 × 2 mm) were freeze-dried to remove excess water for further analysis. The samples were analyzed using an accessory of Attenuated Total Reflection (ATR). The FTIR spectra were recorded in transmittance units within the 4000–400 cm^−1^ range.

The identification of key functional groups was confirmed through the analysis of vibrational patterns in gelatin bonds. Additionally, the nature of the interaction between chitosan and gelatin was explored, providing insights beyond a mere assessment of chemical composition. Characteristic chitosan bands were observed at 3352 cm^−1^, corresponding to the N–H bond of a primary amine and O–H, 1591 cm^−1^ for vibrations of the N–H bond, and 1354 cm^−1^ for methylenes, with bands at 1027 cm^−1^ corresponding to C=O bonds. Gelatin displayed distinctive bands at 1633 cm^−1^, 1521 cm^−1^, and 1229 cm^−1^, indicative of amide groups, given its proteinaceous nature (Figure 2).

### 3.3. Gelatin–Chitosan Hydrogel Swelling Capacity

**Figure 3:**
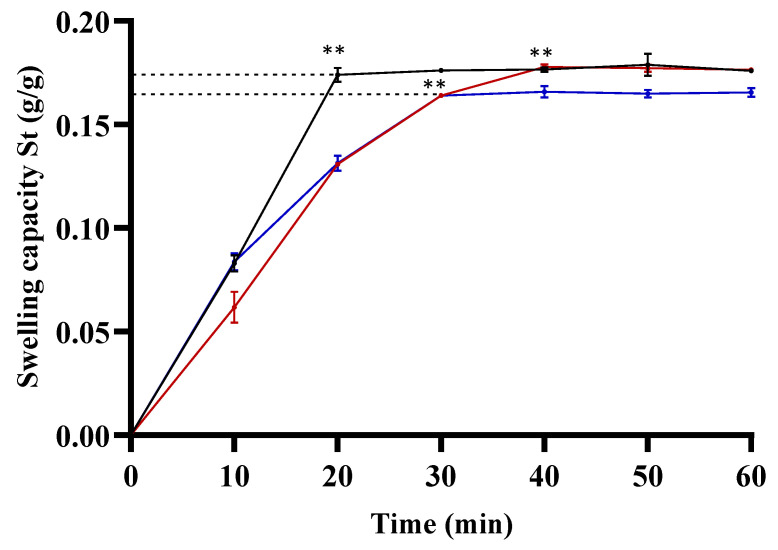
Swelling capacity of gelatin–chitosan hydrogels. Samples (5 × 2 mm) were prepared, weighed, and immersed in 1 mL of distilled water in Eppendorf tubes at 37 °C. Incubation was interrupted every 10 min up to 60 min for weight measurements of the hydrogel samples. The black line corresponds to the control group (gelatin hydrogel), while the red and blue lines represent the experimental group with gelatin–chitosan hydrogel at 0.2% and 0.5%, respectively. ** *p* < 0.01, ANOVA post hoc Tukey test. Each value represents the mean ± SD of triplicate assays (*n* = 9). SD = standard deviation.

The gelatin hydrogel demonstrated a quicker swelling capacity (0.17 ± 0.7 g/g) at 20 min compared to the experimental gelatin–chitosan hydrogels at 0.2% and 0.5%. The absorption capacity reached equilibrium at 40 min (0.17 ± 0.6 g/g) and 30 min (0.16 ± 0.7 g/g), respectively. Figure 3 depicts time (t) on the Y–axis and the St value on the X–axis, representing swelling now until reaching equilibrium, i.e., the water absorption at infinite time or the maximum water retention capacity.

### 3.4. SCAPs Characterization

**Figure 4:**
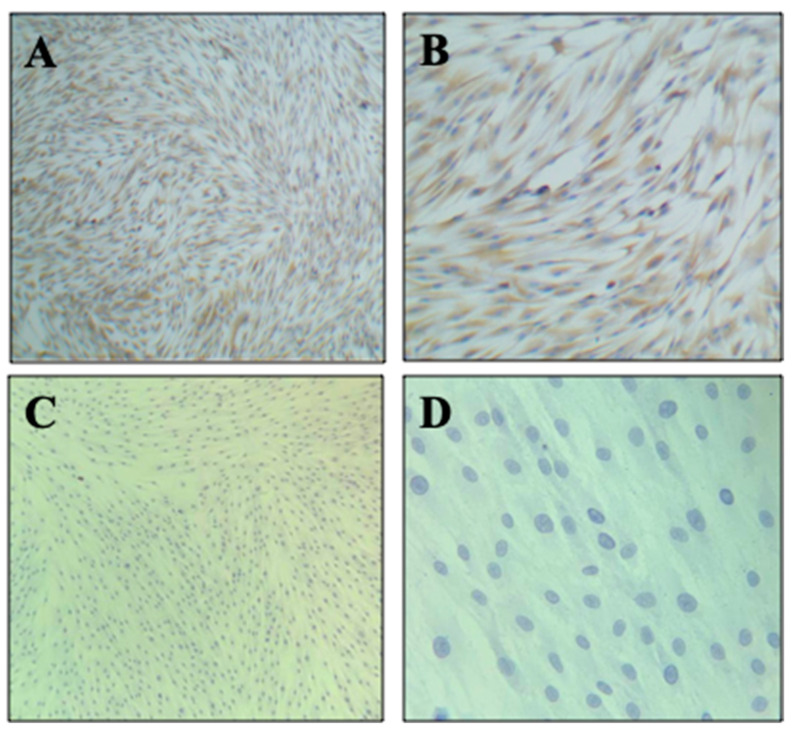
Immunohistochemical characterization of the primary culture of SCAPs was performed. SCAPs at 90% confluence and at passage 5 were cultured for 48 h. Microphotographs (**A**,**B**) illustrate a robust positive vimentin staining, while microphotographs (**C**,**D**) exhibit a negative staining for CD 56, at 20× to (**A**,**B**) and 40× to (**C**,**D**). SCAPs = stem cells from apical papilla; PDL = population doubling level.

Figure 4 displays microphotographs acquired post-immunocytochemistry, revealing strong positive staining for vimentin (Figure 4A,B) and negative staining for CD 56 (Figure 4C,D). This confirms the mesenchymal origin of the SCAPs primary cell culture.

### 3.5. Gelatin–Chitosan Hydrogel SCAPs Interaction and Proliferation

**Figure 5:**
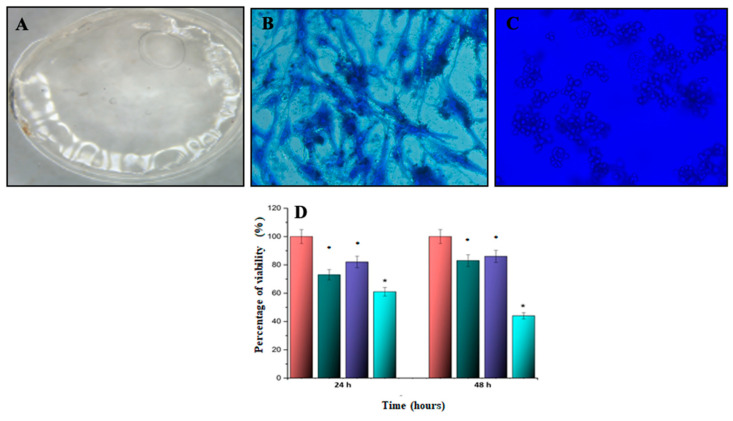
Gelatin–chitosan hydrogel SCAPs interaction and proliferation. The gelatin–chitosan hydrogel (**A**) appearances. SCAPs were subcultured at 1 × 10^6^ cells/mL in a cell culture plate ((**B**), control) or onto the hydrogel for hour to assess cell–hydrogel interaction (**C**) observed in phase contrast microscope or further proliferation at 37 °C with a 5% CO_2_ concentration and 95% humidity for 24 and 48 h (**D**). The interaction was observed under an optical microscope with blue Coomassie stain at 40× magnification. The groups corresponded to negative control (Red), gelatin (Dark Green), gelatin–chitosan 0.2% (Purple), and gelatin–chitosan 0.5% (Green). The relative viable cell number was assessed through the MTT assay. * *p* < 0.05, ANOVA post hoc Tukey’s test. Each value represents the mean ± SD of triplicate assays (*n* = 9). Absorbance at 570 nm. MTT = 3-[4,5-dimethylthiazol-2yl]-2,5-diphenyltetrazolium bromide; SD = standard deviation.

The cell–material interaction exhibits an initial focal adhesion of cells over the hydrogel. Figure 5A,B depict the visual characteristics of the gelatin–chitosan hydrogel and 2D SCAPs cell cultures used as a control. Figure 5C, the cells exhibited a rounded morphology, presenting a distinctive spherical shape. Notably, they displayed well-defined cytoplasm, signifying the integrity of their cellular structure. Furthermore, the cells showed initial focal adhesions, indicative of their attachment to the material and the initiation of intercellular interactions. Subsequently, the proliferation test (Figure 5D), building on the earlier observed cell–material interaction characteristics, revealed that SCAPs exhibited notably greater viability (*p* < 0.01) on the 0.2% hydrogels in contrast to the 0.5% deacetylated gelatin–chitosan hydrogel group at 24 h. Continuing to 48 h, a further increase in cell viability (*p* < 0.05) was observed for the 0.2% hydrogel group, indicating a favorable response to the material.

With respect to the interaction of the cells with the hydrogel, after an hour of interaction, the SCAPs with the hydrogels of both the control group and the study group presented a large number of cells that had adhered to their surface, highlighting that those of the control group and those of the group of 0.2% have a greater density in comparison with the deacetylated hydrogel group at a concentration of 0.5% and with the control group of cells without hydrogel (Figure 5).

### 3.6. Gelatin–Chitosan Hydrogel Antibacterial Effect

**Figure 6:**
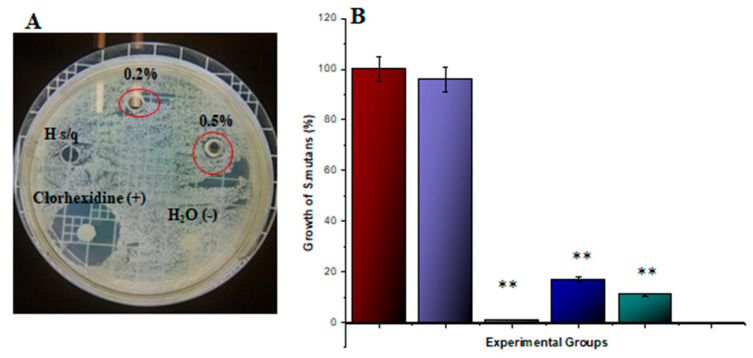
Antibacterial effect of gelatin–chitosan hydrogels. *Streptococcus mutans* (ATCC 36668) were cultivated at 0.5 on the McFarland scale for agar diffusion test (**A**) or microdilution test (**B**). The hydrogels at different concentrations 0% (Purple), 0.2% (Blue), 0.5% (Green) were inoculated. The controls correspond to negative control (Red), positive control (chlorhexidine 0.12%, Black). The inhibition halos were measured in millimeters, and the number of surviving bacteria was determined using the MTT assay. Each value represents the mean ± SD of triplicate assays (*n* = 9). ** *p* < 0.01 ANOVA post hoc Tukey test. Absorbance at 595 nm. ATCC = American type of cell culture; MTT = 3-[4,5-dimethylthiazol-2yl]-2,5-diphenyltetrazolium bromide; SD = standard deviation.

In Figure 6A, regarding the antimicrobial agar diffusion effect, a slight inhibition zone is noticeable for both 0.2% and 0.5% gelatin–chitosan hydrogels. However, it is important to note that this effect does not reach statistical significance (*p* > 0.05) when compared to the positive control. Figure 6B presents the results obtained from antimicrobial tests using the gelatin–chitosan hydrogel via microdilution in broth. The results clearly indicate that the gelatin–chitosan hydrogel significantly influences the inhibition of microbial growth compared to the positive control (chlorhexidine 0.12%). *Streptococcus mutans* exhibited susceptibility to the 0.5% gelatin–chitosan hydrogel with 1 ± 0.5% (*p* < 0.01), and to 0.2% with 17 ± 3% (*p* < 0.01) of survivable bacteria mean, signifying a high sensitivity to this compound.

### 3.7. Gelatin–Chitosan Hydrogel Uniaxial Tensile Strength (UTS)

**Figure 7:**
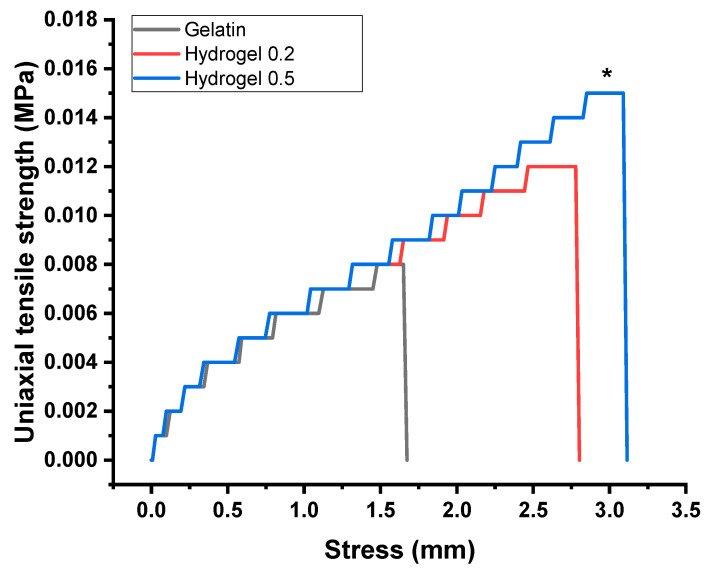
Uniaxial tensile strength (UTS) curves of gelatin–chitosan hydrogels. Gelatin, gelatin–chitosan hydrogel at 0.2%, and 0.5%. The hydrogel samples corresponded to 20 × 10 × 5 mm. The samples were positioned between clamps using a universal testing machine at a cross speed of 1 mm/min until failure occurred. Data were recorded in megapascals (MPa) and stress in millimeters (mm) of *n* = 6 per group. * *p* < 0.01, ANOVA post hoc Tukey test.

The gelatin obtained a UTS of 0.009 ± 0.0007 MPa, gelatin with 0.2% chitosan obtained 0.012 ± 0.005 MPa, and gelatin with 0.5% chitosan obtained 0.015 ± 0.001 MPa. In the ANOVA, gelatin with 0.5% chitosan showed an increase in UTS with a statistically significant difference (*p* < 0.009) compared to gelatin. However, there was no significant difference when compared with gelatin containing 0.2% chitosan (Figure 7).

## 4. Discussion

### 4.1. Gelatin–Chitosan Hydrogel Preparation

Gelatin–chitosan hydrogels present crosslinked polymer networks with excellent water absorption and the ability to facilitate molecule diffusion. They grant precise control over molecular-level chemical interactions, thus affecting biological responses significantly. These hydrogels fall under two categories: physical and chemical [18]. Physical hydrogels form unstable electrostatic bonds through interactions with both anionic and cationic molecules, providing a high level of control over extension and swelling rates. While they have lower mechanical strength and reduced cytotoxicity [19], the thermal and physical gelatin–hydrogel samples produced here align with prior findings.

The results of this research confirm the acceptance of the hypothesis proposed. Hydrogels were successfully synthesized, demonstrating degradation properties, adequate interaction, proliferation, and antimicrobial effects. Several methods have been outlined for synthesizing gelatin–based hydrogels with chitosan, aiming to preserve and enhance the mechanical properties of chitosan. These properties are associated with water content [20,21]. The method employed in this study is based on the technique described by Narvaez-Flores et al. [22] in which concentrations below 0.2% chitosan was used, demonstrating that this is a safe and straightforward technique for manufacturing gelatin–chitosan hydrogels. Furthermore, the hydrogels investigated in these studies maintained their mechanical properties, along with biocompatibility, biodegradability, and eco-friendliness by utilizing recycled raw materials [23,24,25].

### 4.2. Gelatin–Chitosan Hydrogel Degradation

In accordance with previous studies, the degradation of chitosan hydrogels in combination with glycol using the enzyme lysozyme with penicillin–streptomycin resulted in slow degradation, which hindered complete tissue regeneration [26,27]. Another evaluation using chitosan hydrogel with glycol methacrylate in combination with lysozyme demonstrated an accelerated degradation of the hydrogel due to an increase in enzyme concentration [28]. Moreover, some authors have found that degradation can be adjusted by selecting the concentration of crosslinking agents, with higher concentrations leading to longer degradation times. Those results concur with our findings obtained in the degradation assay, which show longer degradation times for hydrogels with higher concentrations of deacetylated chitosan [29]. Here, the gelatin–chitosan hydrogel is resorbable, avoiding the need for a second surgical intervention and reducing the risk of complications.

### 4.3. Gelatin–Chitosan Hydrogel Characterization Using FTIR

In the hydrogels at 0.2% and 0.5%, the same bands can be observed which indicates that regardless of the concentration, the interaction of the gelatin with the chitosan was the same. The wide band in the range of 3200 to 3400 cm^−1^ approximately corresponds with hydrogen-bonded –OH in the hydrogels [30]. The band close to 1644 cm^−1^ could be due to the cross-linked gelatin as this band coincides with what was reported by Matica, et al., in 2017 [31]. The bands close to 1500 cm^−1^ are attributed to the bonds N-acetyl, and bonds observed at between 1230 and 1236 cm^−1^ are attributed by vibrations in the CN group [32].

### 4.4. Gelatin–Chitosan Hydrogel Swelling Capacity

Hydrogels, polymers with water–absorbing capabilities, were assessed for swelling rate by measuring absorption capacity over time. The control, gelatin hydrogel, displayed rapid swelling due to its inherent hydrophilic properties. In contrast, experimental groups with 0.2% and 0.5% chitosan exhibited a slower swelling capacity, suggesting chitosan’s inhibitory influence. The observed phenomenon may stem from specific interactions between gelatin and chitosan, impacting water accessibility within the hydrogel structure. The swift swelling observed in the gelatin control group highlights its hydrophilic nature, primarily constituted by amino acid chains, the fundamental components of proteins [33]. This inherent quality allows gelatin to rapidly absorb and retain water, forming a hydrogel, consistent with its behavior in aqueous environments. Porcine Type A gelatin, a natural polymer derived from pig skin and bones [34], possesses functional groups derived from collagen’s amino acids, including Amino, Carboxyl, Hydroxyl, and Amide groups [35,36]. In the experimental groups with 0.2% and 0.5% gelatin–chitosan hydrogels, the presence of chitosan likely influences gelatin’s swelling pattern. Chitosan, recognized for its biocompatibility and slight hydrophobicity [37], may form a surface layer on gelatin, hindering direct water entry and contributing to a more gradual swelling compared to gelatin alone [38,39]. Additionally, chitosan’s gel–forming capacity in the presence of ions, as found in aqueous solutions [40], may impact water availability for gelatin, limiting absorption and leading to more controlled swelling. These intricate interactions deepen our understanding of the dynamic behavior of gelatin–chitosan hydrogels.

### 4.5. SCAPs Characterization

Vimentin was selected as the antigen to identify the mesenchymal characteristics of SCAPs, as it is described as an omnipresent marker for intermediate filaments of MSCs. The results presented in this study indicate the expression of this protein in SCAPs in vitro cultures, allowing us to classify them as MSCs. The protein exhibits homogeneous distribution and high intensity which is consistent with the findings reported by Kovách et al. in 2021, where the same markers were used to characterize MSCs from follicles, ligaments, and dental pulp [27]. CD 56 antigens, which represent potent markers for cancerous MSCs in tumor progression and metastasis, were used as the control and were found to be negative in the SCAPs samples used in this study [41,42,43].

### 4.6. Gelatin–Chitosan Hydrogel SCAPs Interaction and Proliferation

Previous studies evaluating the cytotoxicity have reported that chitosan at 0.19% and 0.2% displayed cell viability of 89%, indicating no cytotoxicity. They also proposed that concentrations exceeding 0.19% and 0.2% might result in decreased viability upon direct contact with human dental pulp cells (HPC) and human gingival fibroblasts (HGF) [23,24,43]. These findings also coincide with the observations in this study, where the 0.2% gelatin–chitosan hydrogel exhibited no cytotoxicity during the 24 and 48 h periods (cell viability of 82% and 92%, respectively), while the 0.5% group showed moderate cytotoxicity at 24 h (cell viability of 61% and 56%, respectively). Despite the smooth, pore-free surface of the hydrogel, the phase–contrast microscopy images lead to cell adhesion. A higher quantity of adhered cells is observed in the control group and the 0.2% group compared to SCAPs on the culture plate. These findings also correspond with studies that demonstrate cell adhesion and viability at 24, 48, and 72 h through microscopy and evaluate their spherical morphology [44].

### 4.7. Gelatin–Chitosan Hydrogel Antimicrobial Effect

The antibacterial activity is known due to the positively-charged ions disrupting the hydrogen bonds among chitosan molecules, leading to their dissolution in water. Chitosan’s water solubility is significantly influenced by its molecular weight and deacetylation. Removing some acetyl groups enhances water solubility while also improving biodegradability, biocompatibility, and antibacterial effects. It disrupts cells by displacing Ca++ ions from the anionic sites within the membrane [45]. Previous studies have reported that chitosan hydrogels incorporated with lysozyme against *S. aureus* exhibited greater antimicrobial effects than against *E. coli*, attributed to the initially prolonged delay in bacterial growth. Other studies have shown notable antimicrobial effects due to the positively-charged amino acid groups and quaternary ammonium groups that could damage bacterial cell walls. A decrease in chitosan concentration in the hydrogels led to reduced antimicrobial activity [46]. The findings from these studies align with our results, as we observed minimal antimicrobial effects at gelatin–chitosan hydrogel concentrations of 0.5% and 0.2%. A stronger effect was noted in the group with a higher concentration.

### 4.8. Gelatin–Chitosan Hydrogel Uniaxial Tensile Strength

Several studies have assessed the uniaxial tensile strength of hydrogels. Fan et al. in 2016 reported that the tensile strength of the chitosan (CS)/Gel/PVA hydrogel, at various CS/Gel ratios, reached its peak at 2.2 MPa with a CS/Gel ratio of 1:3. In comparison to the Gel/PVA hydrogel, the tensile strength of the CS/Gel/PVA hydrogel showed improvement [47]. Nevertheless, the research also revealed a decrease in both the tensile strength and elongation of the hydrogel with an increase in the CS/Gel weight ratio. This trend can be explained by the added chitosan, which heightened the crosslinking density of the polymer molecules, thereby improving the mechanical properties of the hydrogel. As a result, the tensile strength exhibited improvement to a certain extent. As the CS/Gel weight ratio increased, the hydrogel became harder and more brittle, which led to a decrease in tensile strength. In comparison with our results, chitosan at 0.5% increased the strength. This is consistent with what Nguyen et al. and Fan et al. reported in 2012 and 2015, stating that the crosslinked hydrogel with chitosan, compared to the non-crosslinked variant, showed an increase in ultimate tensile strength (UTS) [48,49]. The hydrogels limitations can exhibit a broad range of mechanical properties, which could influence their potential as solid scaffolds. These materials are highly porous and permeable due to their water content, fostering an environment for the rapid diffusion of oxygen and nutrients. However, their low mechanical strength, particularly in the case of gelatin hydrogels sensitive to temperatures above 40 ºC, presents a challenge.

## 5. Conclusions

The gelatin–chitosan hydrogels exhibited promising attributes, including favorable degradation, notable swelling capacity, mild dose-dependent cytotoxicity, significant proliferation with stem cells from the apical papilla (SCAPs), substantial antimicrobial efficacy against *S. mutans*, and enhanced mechanical properties. These comprehensive findings underscore the potential applications of these hydrogels as postoperative care dressings, showcasing their versatility and effectiveness in various biomedical contexts.

## Data Availability

Data are contained within the article.
